# *Notes from the Field:* HIV Testing in Health Care Facilities — Lesotho, 2017

**DOI:** 10.15585/mmwr.mm6726a5

**Published:** 2018-07-06

**Authors:** Tony Isavwa, Mosilinyane Letsie, Puleng Ramphalla

**Affiliations:** ^1^Elizabeth Glaser Pediatric AIDS Foundation, Washington, D.C.; ^2^Lesotho Ministry of Health; ^3^Division of Global HIV and Tuberculosis, Center for Global Health, CDC.

Lesotho, a small mountainous country surrounded by South Africa, has a population of approximately 2 million persons and an estimated annual income of $1,210 per capita; 73% of the population resides in rural areas (*1*). Lesotho has a generalized human immunodeficiency virus (HIV) epidemic (*2*). During 2016–2017, the prevalence of HIV among persons 15–59 years of age was 25.6%, with an incidence of 1.5 new infections per 100 person-years of exposure (*3*). As the leading cause of premature death in Lesotho, HIV, including acquired immunodeficiency syndrome (AIDS), has contributed to Lesotho having the shortest life expectancy at birth among 195 countries and territories (*4*). Antiretroviral therapy (ART) coverage among persons living with HIV is estimated to be 69.6% (*3*).

Measures to achieve the Joint United Nations Programme on HIV and AIDS (UNAIDS) targets of 90% of all persons with HIV infection knowing their HIV status, 90% of all persons with diagnosed HIV infection receiving sustained ART, and 90% of all persons receiving ART achieving viral suppression (90–90–90) (*5*) have been hampered, in part, by an inability to identify undiagnosed persons with HIV infection. During 2016–2017, 77.2% of persons with HIV infection in Lesotho knew their status (*3*).

The President’s Emergency Plan for AIDS Relief (PEPFAR) supports HIV testing services in 121 health care facilities (113 health centers and eight hospitals) in five of Lesotho’s 10 districts. The five PEPFAR-supported districts account for approximately 75% of all HIV-positive persons in the country (*6*). During the last full fiscal year for which data were available (October 1, 2016–September 30, 2017), a total of 567,062 (70.7%) of 801,654 tests supported by PEPFAR were conducted in these 121 health care facilities.

During May 1–September 30, 2017, a total of 414,907 persons attended outpatient departments in selected PEPFAR-supported health care facilities, including 64,537 (15.6%) who had previously tested HIV-positive and 189,864 (45.8%) who had tested negative within the preceding 3 months, leaving 160,506 (38.7%) persons eligible for HIV testing. Among these persons, 135,563 (84.5%) consented to testing, which identified 6,759 (5.0%) persons with newly diagnosed HIV infection. Thus, 389,964 (94.0%) persons attending these outpatient departments knew their HIV status (including 71,296 [18.3%] who were HIV-positive) before leaving the facility.

Similarly, among 5,927 persons admitted to the eight PEPFAR-supported hospitals during this period, 1,029 (17.4%) patients had previously tested positive for HIV, including 133 (7.9%) of 1,687 children aged <15 years and 896 (21.1%) of 4,240 persons aged ≥15 years. In addition, 3,534 (59.6%) admitted patients had tested negative for HIV during the previous 3 months, resulting in 1,364 hospitalized patients being eligible for testing during their admission. Among these, 1,298 (95.2%) consented; 120 (9.2%) persons tested positive, including 21 (4.0%) of 526 children aged <15 years and 99 (12.8%) of 772 persons aged ≥15 years. Hospital-based HIV testing resulted in 5,861 (98.9%) hospitalized patients knowing their HIV status before discharge, with 1,149 (19.6%) being positive. Positivity rates ranged from 9.2% (154/1,673) among children aged <15 years to 23.8% (995/4,188) among persons aged ≥15 years.

Lesotho has achieved close to 100% HIV testing coverage among hospitalized patients at PEPFAR-supported facilities and is approaching this level among patients seen in selected outpatient departments. In both facility-based testing and community-based testing (i.e., testing done outside health care facilities), testing is being expanded to reach family members and intimate contacts of HIV-positive persons and to promote self-testing (*6*). For Lesotho to achieve epidemic control, all health care facilities need to achieve high HIV testing services coverage. Measures to increase testing for groups with historically poor coverage, including men and adolescents, are needed (*7*). Some strategies include establishment of clinics for men, adolescent corners in existing health care facilities, and expansion of community-based HIV testing to reach and cater to the unique needs of underserved populations.
